# Sialyl-Tn serves as a potential therapeutic target for ovarian cancer

**DOI:** 10.1186/s13048-024-01397-1

**Published:** 2024-04-02

**Authors:** Linah Al-Alem, Jillian M. Prendergast, Justin Clark, Bianca Zarrella, Dominique T. Zarrella, Sarah J. Hill, Whitfield B. Growdon, Venkatesh Pooladanda, David R. Spriggs, Daniel Cramer, Kevin M. Elias, Rawan I. Nazer, Steven J. Skates, Jeff Behrens, Daniel T. Dransfield, Bo R. Rueda

**Affiliations:** 1 Vincent Center for Reproductive Biology, Department of Obstetrics and Gynecology, Massachusetts General Hospital, Boston, MA 02114 USA; 2 Obstetrics, Gynecology and Reproductive Biology, Harvard Medical School, Boston, MA 02115 USA; 3 Siamab Therapeutics, Inc, Newton, MA 02458 USA; 4 Division of Gynecologic Oncology, Department of Obstetrics and Gynecology, Massachusetts General Hospital, Boston, MA 02114 USA; 5 Department of Medical Oncology, Dana-Farber Cancer Institute, Boston, MA 02215 USA; 6 Division of Molecular and Cellular Oncology, Dana-Farber Cancer Institute, Boston, MA 02215 USA; 7 Department of Medicine, Harvard Medical School, Boston, MA 02115 USA; 8 Division of Hematology-Oncology, Massachusetts General Hospital, 55 Fruit St, Boston, MA 02114 USA; 9 Department of Medicine, Massachusetts General Hospital, Boston, MA 02114 USA; 10 Obstetrics and Gynecology Epidemiology Center, Brigham and Women’s Hospital, Boston, MA 02115 USA; 11 Division of Gynecologic Oncology, Department of Obstetrics and Gynecology, Brigham and Women’s Hospital, Boston, MA 02115 USA; 12 Biostatistics Center, Massachusetts General Hospital, Boston, MA 02114 USA

**Keywords:** Sialyl-Tn, Ovarian cancer, Companion diagnostic, Targeted therapy

## Abstract

**Background:**

Ovarian cancer remains the deadliest of the gynecologic cancers in the United States. There have been limited advances in treatment strategies that have seen marked increases in overall survival. Thus, it is essential to continue developing and validating new treatment strategies and markers to identify patients who would benefit from the new strategy. In this report, we sought to further validate applications for a novel humanized anti-Sialyl Tn antibody-drug conjugate (anti-STn-ADC) in ovarian cancer.

**Methods:**

We aimed to further test a humanized anti-STn-ADC in sialyl-Tn (STn) positive and negative ovarian cancer cell line, patient-derived organoid (PDO), and patient-derived xenograft (PDX) models. Furthermore, we sought to determine whether serum STn levels would reflect STn positivity in the tumor samples enabling us to identify patients that an anti-STn-ADC strategy would best serve. We developed a custom ELISA with high specificity and sensitivity, that was used to assess whether circulating STn levels would correlate with stage, progression-free survival, overall survival, and its value in augmenting CA-125 as a diagnostic. Lastly, we assessed whether the serum levels reflected what was observed via immunohistochemical analysis in a subset of tumor samples.

**Results:**

Our in vitro experiments further define the specificity of the anti-STn-ADC. The ovarian cancer PDO, and PDX models provide additional support for an anti-STn-ADC-based strategy for targeting ovarian cancer. The custom serum ELISA was informative in potential triaging of patients with elevated levels of STn. However, it was not sensitive enough to add value to existing CA-125 levels for a diagnostic. While the ELISA identified non-serous ovarian tumors with low CA-125 levels, the sample numbers were too small to provide any confidence the STn ELISA would meaningfully add to CA-125 for diagnosis.

**Conclusions:**

Our preclinical data support the concept that an anti-STn-ADC may be a viable option for treating patients with elevated STn levels. Moreover, our STn-based ELISA could complement IHC in identifying patients with whom an anti-STn-based strategy might be more effective.

**Supplementary Information:**

The online version contains supplementary material available at 10.1186/s13048-024-01397-1.

## Introduction

Ovarian carcinoma (OvCa) is the leading cause of death among gynecological malignancies in the United States [1]. It is estimated that in 2022 approximately 19,880 women will be newly diagnosed, and 12,810 women will succumb to the disease [2]. Despite high initial response rates to current treatment strategies, the 5-year survival rate for women with advanced stage ovarian cancer remains less than 50%. These poor outcomes are due, in part, to the lack of biomarkers that allow for early diagnosis coupled with relatively few targetable mutations or genomic alterations that are present in any quantity across the different ovarian adenocarcinoma histologies. Tumor-associated carbohydrate antigens (TACAs) are aberrant glycosylations of proteins or lipids that are hallmark biomarkers of cancer [3]. Our recent work, as well as work by others, suggested that one such TACA, Sialyl Thomsen-nouveau (Sialyl-Tn or STn; also known as CD175s (Neu5Acα2, 6GalNAc O-Ser/Thr)), may be a target, prognostic biomarker or diagnostic marker of interest in OvCa [4–6]. STn is of low abundance in healthy human tissues but is highly expressed in many cancers, including breast, OvCa, bladder, cervical, colon, pancreatic, lung, and others [6–11]. The presence of STn is generally associated with poor clinical outcomes, therapeutic resistance, and immune suppression [7, 9, 12, 13]. Recurrent OvCa, like many other solid tumors, is thought to result, in part, from residual cancer stem cells (CSCs) that survive cytotoxic chemotherapy and serve as seed cells for the tumor resurgence [14, 15]. Cancer stem cells have membrane proteins with altered glycosylation, including ovarian CSCs, which often express STn [5, 14]. The enzyme responsible for creating STn, ST6 N-Acetylgalactosaminide alpha-2,6-Sialyltransferase 1 (ST6GALNAC1), has been reported to facilitate stemness in OvCa cells via the Akt pathway [16]. Since STn can be added as an O-linked post-translational modification to a variety of protein backbones, using antibodies that specifically target the STn glycan independent of its carrier protein could afford the potential to recognize a more comprehensive array of cancer-specific sialylated proteins [4, 6, 17–19] on CSC and non-CSC populations. Therefore, it is possible that using a glycan targeted therapy alone or in combination with standard chemotherapy may be a rationale therapeutic strategy.

To take advantage of this concept, we initially generated a panel of murine monoclonal anti-STn therapeutic antibodies, including clones S3F and 2G12-2B2 as described in Prendergast et al. 2017 [6]. These murine anti-STn antibodies were conjugated to monomethyl auristatin E (MMAE) to generate antibody-drug conjugates (ADCs). These ADCs demonstrated in vitro efficacy in STn-expressing cell lines, including CSCs and tumor growth inhibition in an OvCa cell xenograft tumor model. Exposure to either S3F-CL-MMAE or 2G12-2B2-CL-MMAE reduced OVCAR3-derived xenograft volume in vivo, depleting STn^+^ tumor cells [5]. Thus, targeting STn in ovarian tumors may be an effective clinical strategy; however, additional in vitro and in vivo studies with the humanized anti-STn -ADC antibodies were needed. Additional knowledge on STn prevalence in both tissue and blood (and their correlations) could also be beneficial with these novel antibodies to predict in a preclinical proof-of-concept assay which tumors could be more likely to respond to such anti-STn-ADCs. Lastly, given that our anti-STn antibody is more specific than others tested [6], it was interesting to know if there was any potential that it could augment CA-125 in the diagnosis of OvCa.

To this end, the studies herein demonstrated on-target effects of our human anti-STn-ADC, reduced viability in a subset of high grade serous ovarian cancer (HGSOC) treatment-resistant OvCa PDOs and in PDXs originating from patients diagnosed with HGSOC with a range of STn expression. Our findings also support the concept that circulating STn might serve as a potential biomarker either independently or in combination with immunohistochemistry (IHC) for triaging patients that might respond to an anti-STn-ADC based strategy. Lastly, our STn ELISA by itself did not outperform CA-125 in the detection of HGSOC. However, it did improve the detection of mucinous and clear cell cancers, albeit the numbers were limited.

## Materials and methods

### Reagents

OvCa cell lines OVCAR3, OV90, and SKOV3 cell lines were obtained from ATCC. OVCAR4 was generously provided by Dr. Panos Konstantinopoulos (Dana Farber Cancer Institute, Harvard Medical School, Boston, MA). OVCAR3 cells were cultured in RPMI 1640 medium (Cat #11,875,093, Gibco-BRL, Gaithersburg, MD) supplemented with 10% fetal bovine serum (FBS, Cat #26,140,079, Thermo Fisher Scientific, Waltham, MA), 1X penicillin-streptomycin (Cat # 15,070,063, Thermo Fisher Scientific), and 0.01 mg/ml bovine insulin (Cat #I0516, Sigma-Aldrich, Natick, MA). OVCAR4 cells were cultured in RPMI 1640 supplemented with 10% FBS and 1X penicillin-streptomycin (Life Technologies, Carlsbad, CA). OV90 cells were cultured in a 1:1 mixture of MCDB 105 medium (Cat #117,500, Cell Applications, San Diego, CA) and Medium 199 (Cat #11,150,059, Gibco-BRL) and 10% FBS. These cell lines have been characterized previously [20–22]. SKOV3 Control (SKOV3^CTL^ and SKOV3 cells overexpressing ST6GALNAC1 enzyme which results in elevated STn (SKOV3-ST6GALNAC1), described in Prendergast et al. [6], were grown in McCoy’s media (Cat # 16,600,082, Gibco-BRL) supplemented with 10% FBS and 1X penicillin and streptomycin. All cell lines were maintained at 37˚C in 5% CO_2_. Established cell lines were subjected to human cell identity verification (STR profiling) at Dana Farber Cancer Institute (http://moleculardiagnosticscore.dana-farber.org). All established cell lines were regularly tested for mycoplasma contamination (Lonza MycoAlert® Mycoplasma Detection Kit Cat # LT07-418, Walkersville, MD).

To better understand the unknown levels of STn in serum, it was necessary to confidently determine the levels of STn on a known control protein, to this end we utilized bovine submaxillary mucin (BSM). BSM is a glycoprotein that is rich in STn and can be used to extrapolate unknown sera STn expression. The lot of BSM used SLBH5656V (Sigma-Aldrich, Cat # M3895) was determined to have 14% sialic acids bound (provided by vendor). This is 0.14 µg sialic acid per µg of BSM. Based on the known sialic modifications of BSM we assumed this is 0.14 µg STn per 1 µg of BSM.

### Antibody generation

The generation of the murine and humanized anti-STn and anti-STn-ADC antibodies used in this assay have been described in the literature by Eavarone et al. [4] and Prendergast et al. [6].

### MTT cytotoxicity assay

OVCAR3, OVCAR4, OV90, SKOV3^CTL^, or SKOV3-ST6GALNAC1 cells were seeded in 96-well plates, incubated overnight in the appropriate complete culture medium, then treated with increasing doses of the human anti-STn-ADC at 0, 2.5, 5, 10, and 50 or 100 nM and then incubated for 72 h or 6–7 days. Cell viability was determined by MTT assay (Cat # M6494, Thermo Fisher Scientific), and the percentage was calculated relative to control (vehicle treated) samples using the formula = (OD sample / OD of control average) × 100.

### Flow cytometry

Flow cytometry was used to assess STn levels in cell lines and primary tumor cells. Following trypsinization, or tissue processing, and incubation with FcR blocking reagent (Miltenyi Biotec, Bergisch Gladbach, Germany, Mouse, Cat # 13,009,257), cells were stained with highly specific mouse anti-STn antibody (Siamab Therapeutics, Inc., Newton, MA) directly conjugated to Alexa Flour 647 using the Zenon antibody labeling kit (Thermo Fisher Scientific Cat # Z25108) or labeled with Alexa Flour 488 (Invitrogen, Carlsbad, CA, Cat # A11017). Fixable Live/Dead Violet (Thermo Fisher Scientific Cat # L34955) was used to determine the level of live and dead cells to exclude dead cells from the staining analysis. After washing, cells were fixed in 4% paraformaldehyde for 20 min, washed and reconstituted in PBS, and then analyzed using Guava Millipore (Millipore, Burlington, MA) or LSRII). Data were analyzed using FlowJo software (version 10.0.8). For analysis of cells derived from xenograft tumors, PDX tumor cells were stained with H^2^k^D^ (BD Biosciences, Franklin Lakes, NJ) to exclude mouse cells from the sample at the time of analysis and only determine levels of STn expression in human cells.

### In vivo treatment studies

All mouse studies were carried out in compliance with our Institutional Animal Care and Use Committee guidelines at Massachusetts General Hospital. For establishment of PDXs, tumor cells originally derived from patients (who provided informed consent) diagnosed with HGSOC were processed to homogenized single cell suspension and re-suspended in PBS: Matrigel ® (Cat # 354,234, Sigma-Aldrich) (1:1) and subcutaneously (s.c.) injected into ∽ 8-week-old female NOD/SCID mice (Jackson Laboratory, Bar Harbor, ME). Once initial tumor xenografts were formed, they were harvested for histological verification and tumor was cryopreserved for future rederivation. For the present study cryopreserved tumor samples were thawed and implanted in mice and allowed to form. Once sufficient tumor burden was formed the tumors were harvested, mechanically and enzymatically processed to remove mouse cells and an equal number of tumor cells were resuspended and injected into immunocompromised mice as described above and previously [23–25].

All animals were monitored regularly for tumor formation, and tumor volume was calculated using the formula (length x width x height)/2 as has been described [26]. When tumor volumes averaged between 200 and 250 mm^3^ the mice were randomized into the different arms to have a similar average tumor volume in each arm (a minimum of 4 mice/arm). Mice were treated with either vehicle control, isotype-ADC, human anti-STn-ADC, carboplatin/paclitaxel (Cat # C2538/ Cat # T1912 Sigma Aldrich) or a combination thereof, depending on the experimental design. Human anti-STn-ADC was administered at 5 mg/kg in sterile saline weekly as determined by our previous pharmacokinetic studies [4]. Similarly, the animals treated with isotype-ADC were also treated at 5 mg/kg in sterile saline weekly. Carboplatin and paclitaxel treatments were at 25 mg/kg and 12 mg/kg, respectively, weekly. Carboplatin and paclitaxel dose concentrations were based on our prior studies [23, 25]. Tumors were measured every 3 to 4 days with calipers, and mice were weighed twice a week. If mice lost more than 15% of their body weight or tumors grew to exceed the preset limitations, they were sacrificed. At the study’s completion, tumor samples from some xenografts were formaldehyde-fixed and paraffin-embedded for STn staining.

### Organoid culture and treatment

Organoids were cultured as described previously [27]. All organoids were tested for mycoplasma (MycoAlert® Kit) and all were negative. For sensitivity analysis, organoids were digested with TrypLE (Life Technologies Cat #12,604), plated in 20% Matrigel, and then treated with either human anti-STn-ADC or Isotype-ADC at 0, 0.02, 0.05, 0.1, 0.15, 0.2, or 0.25 nM and read by CellTiter-glo (Cat # G7572, Promega, Madison, WI) six days later. On day 1, four wells were read with CellTiter-glo for growth rate correction. Growth rate corrected dose curves and area over the curve were calculated as described [28].

### Patient selection and data

Blood samples were collected from patients with either OvCa or benign masses and processed for serum. Patient consent forms and sample collections were approved and in compliance with the Institutional Review Board guidelines (Protocol #s 07–049, 2016P002742 and 2000P001678). Research coordinators double-coded the samples, and the samples were run blindly. At the end of the study, the code was revealed, and results analyzed as described in the statistical analysis section. Patient characteristics are shown in Supplemental Table 1. Four hundred twenty serum samples were obtained from the VCRB MGH Gyn sample repository. All samples that were used for diagnostic assessment were collected within five days prior to surgical debulking. Clinical CA-125 values were obtained from the patient’s chart. If there were multiple CA-125 values, the results closest to the time serum was collected before surgery were used for comparison. A representative tumor piece was fixed in paraformaldehyde for a subgroup of patients and used for immunohistochemistry studies described below. Clinical correlates such as histologic subtype, stage, grade, first line treatment, number of treatment cycles, and response to treatment were collected (see Supplemental Table I).

Independent of the diagnostic potential, we initiated a preliminary experiment to assess what impact surgical debulking had on circulating STn levels. For this we obtained a set of matched blood samples from patients diagnosed with HGSOC prior to surgery and again post-surgery and subjected them to our ELISA as described.

### Immunohistochemistry (IHC)

A subset of paraffin-embedded ovarian tumor samples from patients with matched blood sampled were sectioned at 5 μm thickness to compare tissue levels of STn with the patients corresponding serum STn levels. The subset of matched samples included 41 serous, 10 mucinous, 12 clear cell, and 4 endometrioid histologies. Antigen retrieval was performed using 10nM sodium citrate solution at 120º C using a pressure cooker for 15 min. Tissues were incubated with 3% H_2_O_2_ (Cat # S25359, Thermo Fisher) for 20 min, then blocked with 6% serum cocktail (normal Horse Cat # S2000, Bovine Cat # SP5050, Goat Cat # S1000 serum from Vector Labs, Burlingame, CA) for 20 min. Tissues were then incubated with either murine anti-STn primary antibody or control mouse antibody MOPC isotype at 10 µg/mL (MOPC173, Biolegend, San Diego, CA), or no primary antibody diluted in the blocking cocktail. After washing with PBST, slides were incubated for 45 min with anti-mouse antibody (Santa Cruz, Dallas, TX). To visualize the staining, 3,3’-diaminobenzidine (DAB) was used (Cat # SK-4100; Vector stains, Burlingame, CA). Slides were counterstained with hematoxylin (Cat # CS402-1D Thermo Fisher Scientific) and Scott’s water. Finally, slides were dehydrated and mounted with coverslips. Membrane STn positivity was scored by a board-certified pathologist (SJH) blinded to the sample code. A score of 0 meant minimal membrane staining with STn in less than 5% tumor cells, or no staining with STn was identified. A score of 1 indicated minimal to moderate STn membrane staining in 5–24% of tumor cells. A score of 2 indicated moderate to strong membrane staining in 25 − 50% of tumor cells. A score of 3 indicated moderate to strong membrane staining in greater than 50% of the tumor cells.

### Development of an STn biomarker ELISA screening assay

To develop a sandwich ELISA to identify STn expression in patient serum, we utilized commercial, and Siamab generated murine anti-STn antibodies [4, 6] in a research use only (RUO) grade assay. A subset of these anti-STn antibodies was chosen for sandwich ELISA testing, and pairing was guided based on their light chain CDR sequences. Proof of concept studies matched murine and humanized antibody pairs. The pairing included murine S3F, 2G12-2B2, CC49, 5G2-1B3, B72.3, and humanized Hu3F1 L1H1, Hu2G12-2B2 L0H3, Hu5G2-1B3 L1H2 for testing along with isotype controls in a matrix format. Isotype controls included were murine MOPC173 (BioLegend, Cat # 400,264), Plates (Corning, Cat # 9018) were coated with 1, 3, or 5 ug/mL murine antibodies in coating buffer (50 mM sodium carbonate/bicarbonate pH 9.5) overnight at 4 C. After 3x washing with phosphate-buffered saline with 0.05% Tween-20 (PBS-T), plates were blocked with blocking buffer (1% ovalbumin (OVA) in PBS) for 1 h at room temperature. Buffer was removed, and 100 µl/well of STn glycoprotein sample (0.000125 mg/mL BSM, Sigma Aldrich) diluted in blocking buffer was added and incubated for 90 min at 37ºC. Plates were then washed 2x with PBS and a subset of wells had their sialic acids oxidized by treatment with 2 mM periodate. Wells were incubated for 20 min at 4 °C. Plates were then washed 3x with PBS-T, and 3ug/mL secondary humanized anti-STn antibodies were added to the wells diluted in blocking buffer and incubated for 1 h at room temperature. Plates were washed 3x with PBS-T followed by incubation with 0.08 µg/mL peroxidase-conjugated goat anti-human antibody (Cat #109-035-098, Jackson ImmunoResearch, West Grove, PA) for 1 h. Next, wells were washed 3x with PBS-T, and then wells were incubated with 100 µL of enzyme substrate (0.5 mg/mL o-phenylenediamine; 0.03% H_2_O_2_ in citric/phosphate buffer pH 5.5). The enzyme reaction was terminated by the addition of an equal volume of 1.6 M sulfuric acid. Optical Density (OD) readings of periodate and non-periodate treated wells were determined at 490 nm. Binding affinities of the antibody pairs were compared by subtracting the periodate treated wells from the non-periodate treated wells to obtain the periodate-sensitive STn binding.

### Statistical analysis

All experiments were carried out as at least 3 biological replicates and the data were analyzed with GraphPad Prism (GraphPad Software, La Jolla, CA). Patient serum biomarkers were analyzed using the R statistical computing environment (4.0.2) [29] and Stan, a platform for statistical modeling [30] using the R package rstan [31]. Bars represent mean ± SEM. One-way ANOVA was conducted to assess for significant differences seen in tumors treated with human anti-STn-ADC. A statistical significance is determined if the *p* value was < 0.05.

To assess the complementarity of STn to CA-125 as a biomarker for ovarian cancer, bivariate mixture models [32] estimated the joint distribution of STn and CA-125. The underlying distribution was a bivariate *t*-distribution to provide robustness to outliers. A mixture model for one serum biomarker (e.g., CA-125) in cases has a fraction of cases (∽ 80%) that overexpress the biomarker compared to control patients, and the complementary fraction (∽ 20%) that does not overexpress the biomarker, that is, its distribution is the same as the distribution for the control group (benign ovarian disease). A bivariate mixture model for two serum biomarkers in cases has four components, the first where both biomarkers (STn and CA-125) are overexpressed, the second has STn overexpressed but not CA-125, the third has CA-125 overexpressed but not STn, and the fourth where neither biomarker is overexpressed and hence has the same bivariate distribution as the controls. Each component has a fraction of the cases – with the four fractions summing to 100%. The estimate for a fraction of cases in the second component, where STn is overexpressed but CA-125 is not, estimates the complementarity of STn to CA-125.

## Results

### Anti-STn-ADC selectively targets cells that express STn in vitro

**Fig. 1:**
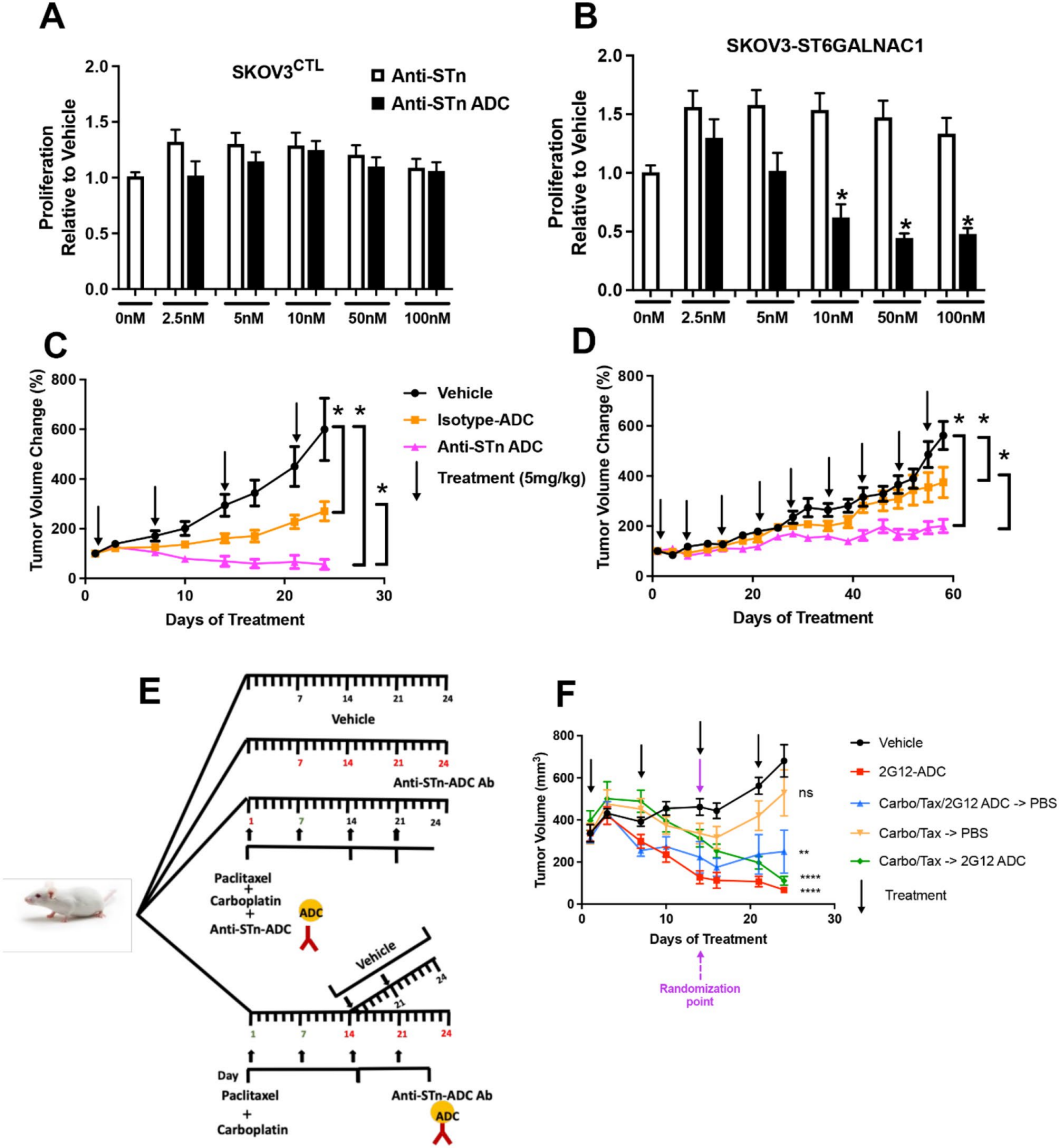
Demonstrating on-target effects and response of humanized anti-STn-ADC in pre-clinical models. SKOV3^CTL^ cells which express little to no STn and SKOV3 cells that were stably transfected with ST6GalNac1 to express elevated levels of STn (SKOV3-ST6GALNAC1) were plated in 96-well plate and then treated the next day with 0, 2.5, 5, 10, 50 or 100 nM of humanized anti-STn ADC (A, B). Seventy-two hours later the metabolic activity as an indirect measure of viability was assessed via MTT assay. The humanized anti-STn-ADC did not affect the SKOV3^CTL^ cells, whereas a dose-dependent decrease in metabolic activity was evident in the SKOV3 cells with elevated levels of STn. Figure 1C, D show the response of 2 independent HGSOC PDX models expressing different levels of STn. Following randomization of mice in each of the arms (n = minimum of 4 mice/arm), they were treated with either vehicle, an isotype-ADC, or anti-STn-ADC weekly at a dose of 5 mg/kg. The graph showing the collective results in mice harboring tumors with high percentage STn positive tumor cells are shown in panel C and those hosting tumor with low a percentage of STn positive tumor cells are shown in panel D. Panel E represents a schematic of the sequential and combination treatment strategy. Panel F represents the results of the multi-arm experiment included one arm receiving vehicle at days 0, 7, 14 and 21 (Black Line); one arm received anti-STn-ADC at day 0, 7, 14 and 21 (Red Line); one arm received the combination of anti-STn-ADC and carboplatin/paclitaxel (on days 0, and 7) which was then followed by weekly PBS (on days 14, 21 Blue Line). One arm beginning with a larger number of mice hosting tumor received carboplatin and paclitaxel (1 x weekly x 2 weeks, days 0, and 7) after which the mice were then randomized across two additional arms. One receiving just PBS on days 14 and 21 (Orange Line) after cessation of carboplatin/paclitaxel (which results in a resurgence of tumor growth). The final sub arm received anti-STn -ADC (on days 14 and 21) following the cessation of the carboplatin/paclitaxel (Green Line) to assess whether it could impede the resurgence. At the end of the experiment all arms had a minimum of 4 mice. Asterisks represent differences (*p* < 0.05) compared to vehicle controls

To determine the on target effects of the humanized anti-STn-ADC (2G12), we utilized the SKOV3 wild type (SKOV3^CTL^) line, which was chosen because it had little to no detectable levels of STn and SKOV3 cells that were stably transfected with ST6GalNAc1 to increase STn on the cell surface (SKOV3-ST6GALNAC1) [6] (Supplemental Fig. 1A, B). There was no significant effect on cell proliferation observed using MTT assays following treatment with humanized anti-STn-ADC of SKOV3^CTL^ cells. However, there was a dose-dependent decrease in MTT activity in SKOV3-ST6GALNAC1 cells (Fig. 1A and B).

### Anti-STn-ADC is more effective in tumors with higher levels of STn expression in vivo.

One of the therapeutic challenges in OvCa is the heterogeneity of the tumor and continual resurgence of tumor cells that become resistant leading to high patient mortality. We postulated that PDXs more faithfully replicate the heterogenous nature of OvCa in patients more so than xenografts generated from many of the commercially available long term established OvCa cell lines. Therefore, we identified two HGSOC PDXs for comparison, one expressing a low percentage of STn positive tumor cells (5.48 ± 0.85 SEM) and one with a higher percentage STn positive tumor cells (53 ± 0.1 SEM), as determined by flow cytometry (Supplementary Fig. 2A and B). Humanized anti-STn-ADC (2G12, 5 mg/kg per week as determined by PK studies [4]) was tested in these two OvCa PDX models. Mice hosting PDX with high STn (Fig. 1C) tumors had a quick and robust response (50% decrease in tumor volume in ∽ 4 weeks) to treatment when compared to mice hosting tumors with lower STn (Fig. 1D). Despite the lower STn expression, there was still a benefit from anti-STn-ADC. Mice were weighed every 3–4 days and treatment had no significant effect on mouse weights in either of the PDX models (Supplemental Fig. 2Cand D).

### STn-ADC was more effective than carboplatin/paclitaxel treatment

To determine whether anti-STn-ADC (2G12) has comparable effects to carboplatin/paclitaxel treatment we utilized the high STn expressing PDX described above (Fig. 1C), examining anti-STn-ADC alone or in combination or sequential treatments. Animals were randomized and treated when tumors reached an average size of 300 mm^3^ (Fig. 1E). Animals were initially randomized to four arms which included vehicle, anti-STn-ADC alone, carboplatin and paclitaxel in combination or sequentially with anti-STn-ADC and carboplatin/paclitaxel alone. At the designated time point (day 14 or third treatment point) the arm receiving carboplatin and paclitaxel in combination with anti-STn-ADC was received vehicle for the remaining treatment points. The mice receiving carboplatin and paclitaxel alone were then randomized to receive vehicle alone or anti-STn-ADC alone (Fig. 1F). The animals treated with anti-STn-ADC alone demonstrated a decrease in tumor size compared to vehicle at the end of week 2. To model impact of anti-STn treatment on tumor resurgence, animals that were treated with carboplatin and paclitaxel for two weeks were then randomized to treatment with vehicle or anti-STn-ADC. Tumors that were switched to vehicle increased in size with time as previously shown [25] and those switched to treatment with anti-STn-ADC continued to decrease.

### Organoid cultures respond to anti-STn-ADC in a dose-dependent manner.

To determine whether using the human anti-STn-ADC could affect tumor cell viability in PDOs, we tested anti-STn-ADC on four PDO lines. The four lines had variable genetic backgrounds and known therapeutic response. Briefly, the PDO 17–39 was derived from a patient diagnosed with recurrent HGSOC. The tumor was reported to have a *BRCA1* germline mutation and a *MYC* amplification. 17–121 was derived from a patient diagnosed with recurrent HGSOC with *MYC* amplification. PDO 17–116 was derived from a patient who had received neoadjuvant chemotherapy following a diagnosis of HGSOC. PDO 18–47 was derived from a patient diagnosed with HGSOC prior to treatment and is *CCNE1* amplified. All PDOs were reported to harbor *TP53* mutations similar to the parent tumor [27].

**Fig. 2:**
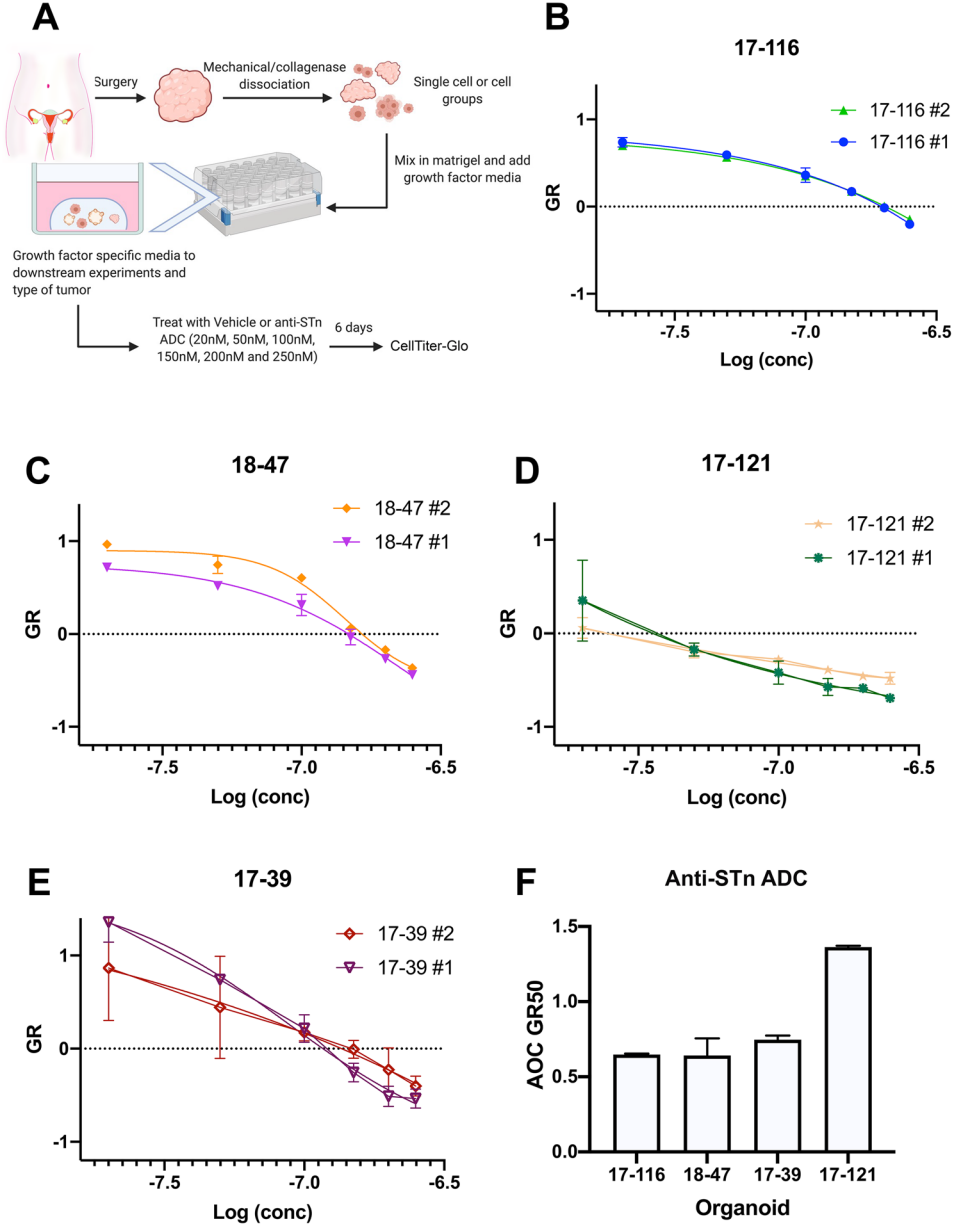
Assessment of anti-STn-ADC effects in a panel of HGSOC PDOs. The PDOs were treated with either vehicle or increasing concentrations of anti-STn-ADC on day 1. Each organoid was treated as two individual repeats for each line and cultured for 6 days (3 replicates per dose in each repeat). An untreated series of wells was read on day 1 to compare to the day 6 treated wells to allow for mathematical growth rate correction (Panel A). Figure 2B, C, D, and E represent the area over the growth rate corrected dose curve (AOC) for each organoid line demonstrating its sensitivity to treatment with anti-STn-ADC compared to its vehicle control. Figure 2F illustrates the AOC GR50 of anti-STn -ADC in each line

Three of the lines were known to be treatment resistant and one of which is still standard of care treatment sensitive [27] (Fig. 2 and Supplemental Figs. 3 and 4). The PDOs were treated with increasing concentrations of anti-STn-ADC for 6 days and proliferation was determined using Cell Titer-glo (Fig. 2A-E). Interestingly, the line most sensitive to anti-STn-ADC, 17–121 is a line that has been determined to be multi-therapy resistant as shown in the area over the curve (Fig. 2F). To determine whether STn levels would be detectable in the media, our ELISA was used, however the levels in organoid culture were below detection. We then assessed the levels of STn in the HGSOC PDOs by flow cytometry using the mouse anti-STn antibody or IgG control. The organoids ranged from 5 to 23% positivity (Supplemental Fig. 3). In line with expectations, the PDO with the highest level of STn (17–121) responded more favorably to the anti-STn -ADC.

### Expression of STn in blinded patient samples

**Fig. 3:**
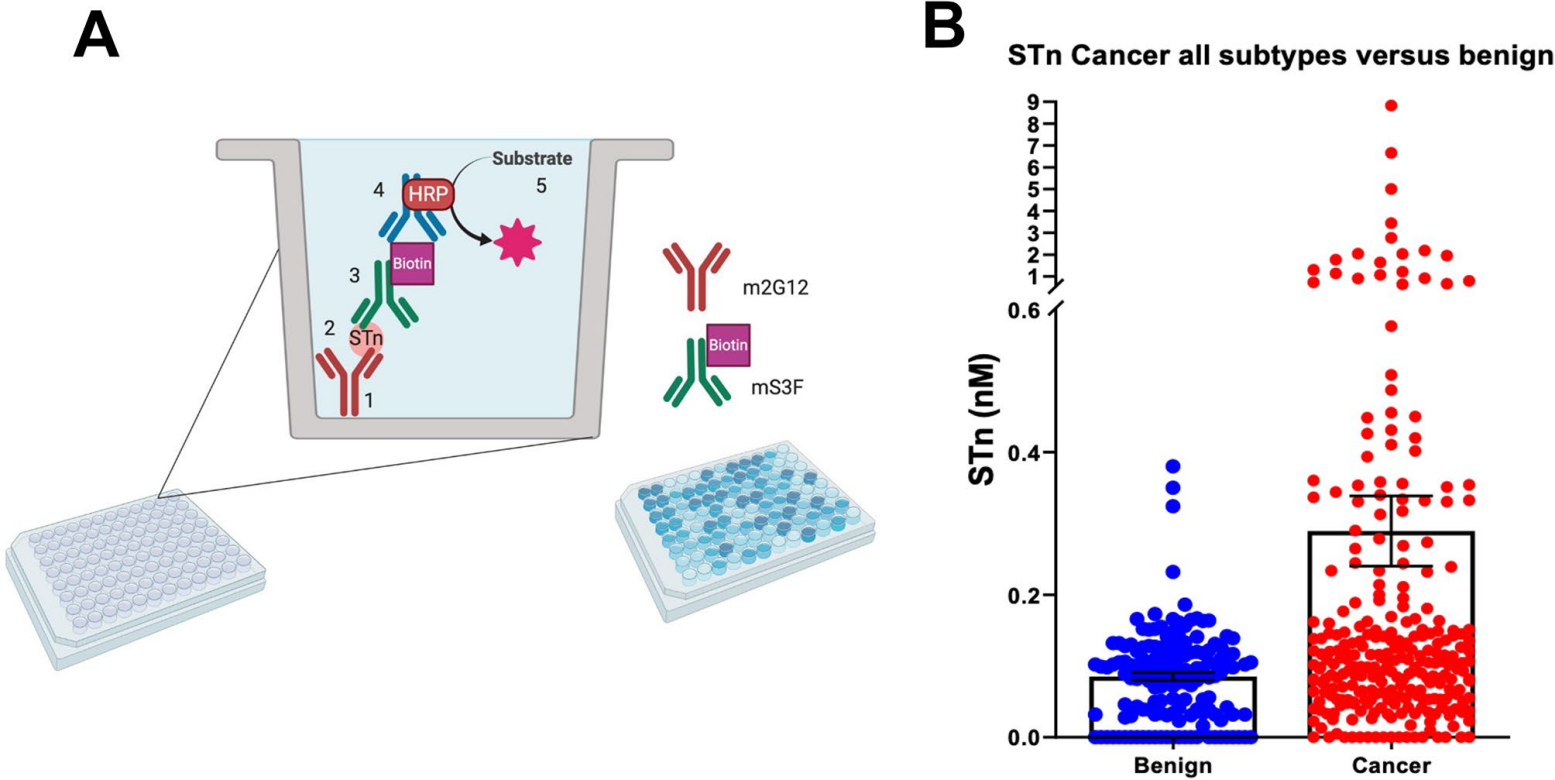
Development and testing of a custom ELISA. Panel A provides a schematic of the ELISA. Panel B illustrates the STn levels in serum collected from patients diagnosed with OvCa (all subtypes) compared to retrospective collected serum collected from patients diagnosed with benign conditions (endometriosis, uterine fibroids, follicular cysts etc.)

Patient serum samples were tested using the custom RUO ELISA developed to determine whether STn serum levels were a successful biomarker in predicting OvCa versus non OvCa patient status (Fig. 3, A and B). A total of 420 samples were run at various dilutions and STn levels were calculated based on the BSM standard curve. Out of these, 274 were from patients with OvCa (Details in Supplemental Table 1), 146 samples were from benign gynecological diagnoses (i.e., endometriosis, endometrioma, follicular cysts, fibroids etc.). On average, benign samples had significantly lower STn levels than cancerous samples (median values: 0.1 vs. 0.3 nM STn p value < 0.05). STn levels were able to determine cancer from non-cancer at a 15.3% sensitivity at 97.1% specificity with a cutoff value of 0.2 nM.

### Correlation between STn and patient clinical outcomes

**Fig. 4:**
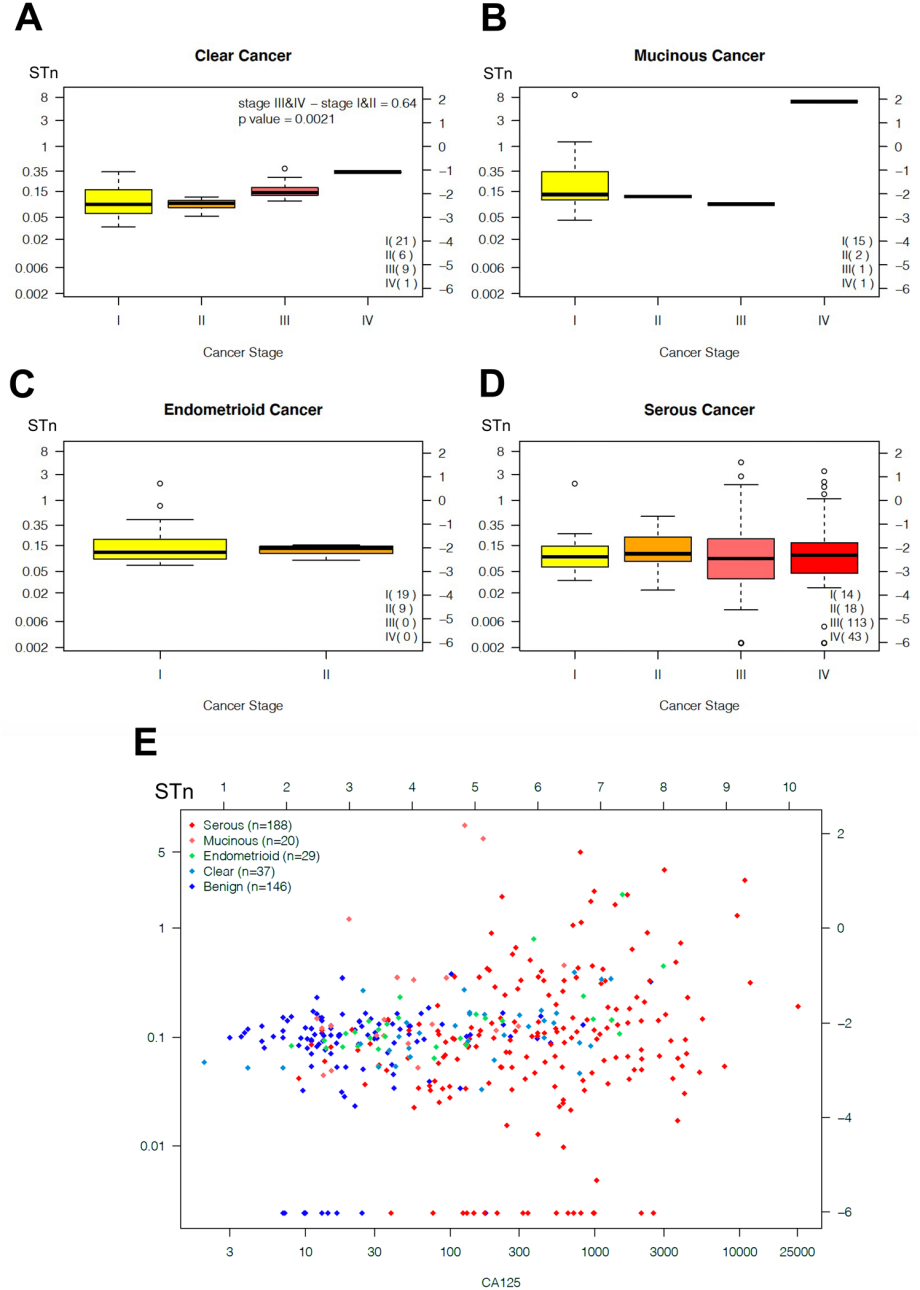
Box and whisker plots displaying distribution of serum STn levels (log concentration scaled left axis, log (concentration) right axis) by stage for clear cell OvCa with significant difference between early stage (I & II) and late stage (III & IV) *p* = 0.002 (panel A), mucinous ovarian cancer (panel B), endometrioid OvCa (panel C), and serous OvCa (panel D). Serum STn versus serum CA125 bivariate plot coded by histology (serous (red), mucinous (orange), endometrioid (green), clear cell (blue), benign (purple) panel E)

Clinical measures and survival outcomes (OS and PFS) were collected for the patients (median 7 years follow-up) and correlative analysis was done using log-rank tests and Cox proportional hazards models. Samples were grouped into clear cell (Fig. 4A), mucinous (Fig. 4B) endometrioid (Fig. 4C), and serous (Fig. 4D). There was no predictive association between serum STn expression and cancer stage in endometroid, serous, nor mucinous cancers. There was a correlation between stage and STn levels in clear cell subtype by *t*-test (*p* = 0.002) with late stage (III & IV) cancers having a median STn 90% higher than early stage (I & II). Future cohorts may benefit from increased patient numbers in specific staged cancers by histology to improve statistical power.

To examine the ability of pre-treatment patient levels of STn expression to predict response to treatment with platinum and paclitaxel based strategy, we grouped the cohort according to clinical response to therapy and performed correlative analysis by *t*-test, and log-rank test and Cox proportional hazards tests for survival outcomes (OS and PFS). These tests showed no predictive power of pre-treatment serum STn for the following outcomes. Patient clinical responses to treatment were determined as sensitive (response > 6 months), refractory (did not respond), resistant (responded < 6 months). There was no statistical difference in pre-treatment STn levels between patients with different responses to chemotherapy (Supplemental Fig. 5A). Kaplan Meier curves for OS and PFS were determined and there was no correlation between pre-treatment STn levels and patient PFS and OS (Supplemental Fig. 5B and C).

### Determining if circulating STn can augment CA-125 as a diagnostic

In comparison, patient CA-125 levels showed 79% sensitivity at 97.1% specificity using a cutoff of 35 IU/mL. There were 25 patients (9.1%) with OvCa but low levels of CA-125. Amongst this group of low CA-125 patients, only 4 showed higher levels of STn, limiting the increase in sensitivity due to STn above the sensitivity provided by CA-125 to about 2%. The combination of serum CA-125 and STn (Fig. 4E) did not improve biomarker prediction of cancer vs. non-cancer. The bivariate robust t-distribution mixture model estimated that combining all histologies the four components had the following estimates for a fraction of cases: (1) STn and CA-125 overexpression: 51.1%; (2) STn overexpression, CA-125 same as controls: 1.6%; (3) CA-125 overexpression, STn same as controls: 25.3%; and (4) STn and CA-125 same expression as control patients: 21.1%. However, for mucinous cases, the four components had the following fractions: (1) STn and CA-125 overexpression: 13.8%; (2) STn overexpression, CA-125 same as controls: 9.3%; (3) CA-125 overexpression, STn same as controls: 21.1% and (4) STn and CA-125 same expression as control patients: 47.7%. Hence for mucinous subtype, serum STn was complementary to CA-125 in 9.3% of cases in our cohort, providing complementarity to CA-125. However, greater numbers of mucinous ovarian cancer cases are required before this indication that preoperative serum STn levels may add predictive power to serum CA-125 levels for distinguishing ovarian cancer from benign pelvic disease and such estimates are deemed stable and accurate.

### STn levels pre and post-surgical debulking.

Currently, OvCa disease progression monitoring is typically accomplished using CA-125 via clinically accessible blood assays. As expected, CA-125 values normally decline post-surgical debulking. The decrease is attributed to the marked reduction in tumor volume, although it is generally appreciated it is not an exact correlation. To date we had no knowledge as to what impact surgical debulking had on circulating STn levels. To begin to address this question an independent set of matched blood samples derived from patients diagnosed with HGSOC prior to surgery and again post-surgery were assessed in our ELISA as described. We determined that circulating STn levels declined post debulking surgery (Supplemental Fig. 6) as has been reported for CA-125. These results, albeit preliminary, suggest that a future study with a larger cohort with more detailed clinical information related to the amount of disease, whether the surgery was sub-optimally or optimally debulked would be of interest. In addition, assessment of circulating levels of STn pre and post-surgery at regular intervals until there is evidence of recurrent disease as determined by elevated CA-125 and or imaging could determine if there is any value for STn as an additional marker of recurrent disease. Whether post-surgical circulating STn levels could inform the onset of tumor resurgence is yet to be determined.

**Fig. 5:**
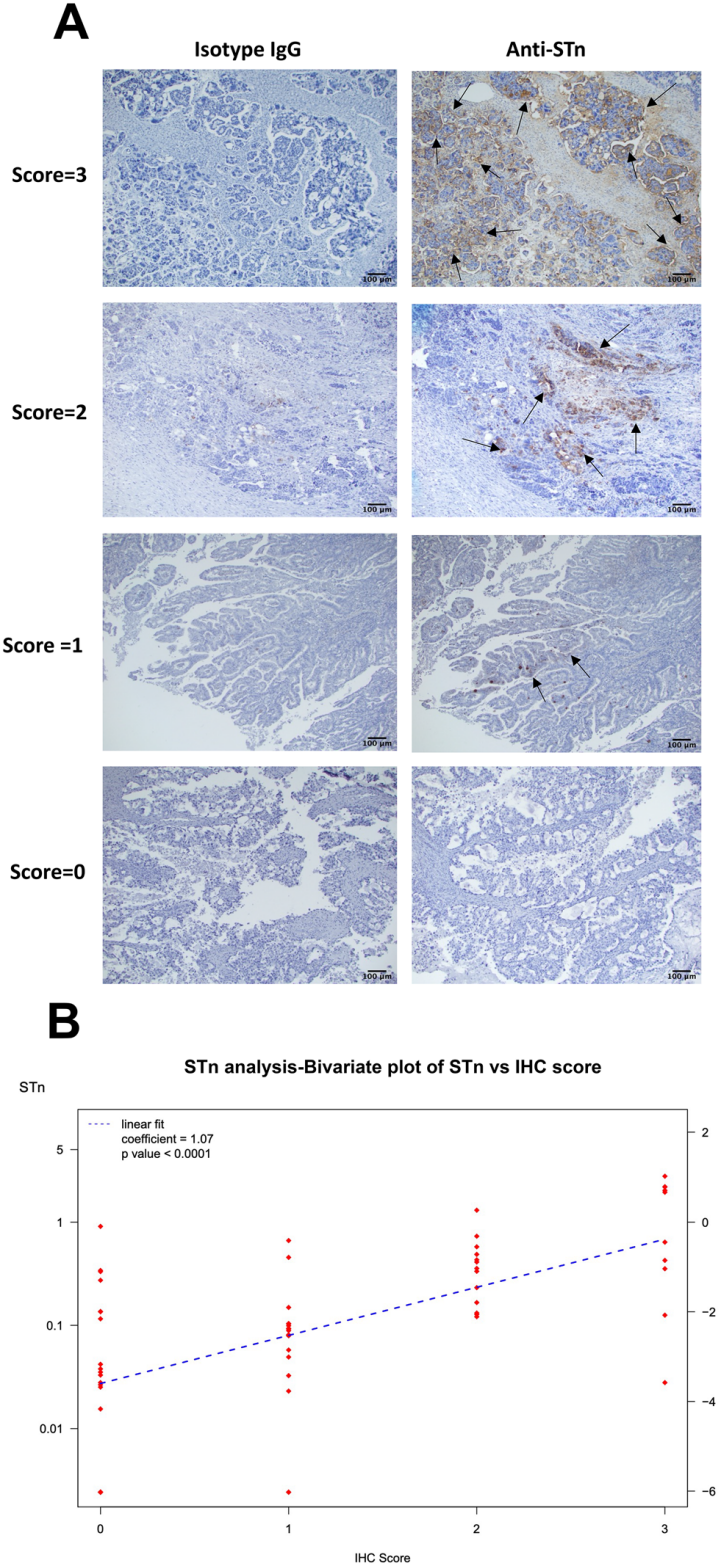
Levels of positive STn staining observed in fixed embedded tumor samples reflected levels of serum STn. Representative examples of IHC scoring scheme (Panel 5 A). Arrows indicate STn positive cells. Scale bars represent 100 μm. A linear regression analysis of the STn serum levels and IHC (0, 1, 2, 3 levels) showed they were positively correlated (Fig. 5B, *p* < 0.0001) with median STn serum (Panel B)

### Immunohistochemical analysis of serum matched tumor samples.

To determine whether the levels of STn observed in tumors mimicked the levels of serum STn, we initially stained a subset of HGSOC (stage III, *n* = 30) samples that were evaluated and scored (scoring scheme examples are shown in Fig. 5A).

A linear regression analysis of the STn serum levels and IHC (0, 1, 2, 3 levels) for all histologies (*n* = 52, including serous, clear cell, mucinous, and endometrioid) showed they were positively correlated (Fig. 5B, *p* < 0.0001) with median STn serum almost tripling with each unit increase in IHC (292% increase). Only two patients (3.8%) had low serum STn, below the median STn of 0.1 nM, with high (level 2 or 3) IHC STn expression. STn can present on a variety of proteins, and we do not expect all to be shed at the same rate. Of note, we observed evidence of heterogeneity regarding the presence of STn positive staining when evaluating different samples collected spatially and temporally apart. This variation highlights that the expression of STn could be heterogenous throughout a patient’s clinical treatment, supporting the argument for having a complimentary analysis via a blood-based assay. Future studies could benefit our understanding of these observed STn biomarker changes, and correlations between tissue and blood STn prevalence, using powered longitudinal cohorts.

## Discussion

The current study demonstrated on-target effect of treatment with a humanized anti-STn-ADC in OvCa cells, HGSOC PDOs, and patient derived serous ovarian cancer xenograft models. This significantly extends our previous assessment of STn expression in some of the long term established ovarian cancer cell lines and xenograft model with the mouse anti-STn-ADC and the more recent investigations characterization of the humanized anti-STn-ADC [4–6]. In addition to the functional studies, we developed an STn ELISA as a proof-of-concept to assess which patients express elevated STn levels and thus might benefit most from an anti-STn based strategy. Unlike other studies [12, 33, 34] we did not observe an increase in circulating STn levels correlating with advanced stage. Moreover, there was no correlation with circulating STn level and PFS or OS in our cohort using our ELISA. There was, however, evidence that the serum levels were reflective of the tumor levels of STn. These differences between our results and that of the others could be attributed in part to the different specificities of the different antibodies used, as previously described [6, 19]. Lastly, while our ELISA could distinguish between malignant and non-malignant samples with a relatively high degree of confidence, the assessment of serum STn using our ELISA did not outperform or augment CA-125 in the detection of serous ovarian cancer. However, it did augment CA-125 in detecting mucinous and clear cell cancers in our cohort, albeit the numbers of samples representing these subtypes were limited and would benefit from the analysis of a larger cohort to have any confidence. Further development of this blood-based ELISA immunoassay platform to improve sensitivity and dynamic range could be beneficial to this unique target. Our data herein suggest the humanized anti-STn-ADC preferentially targets cells that express STn in an in vitro model. This was shown by treating the SKOV3^CTL^ OvCa cell line, which naturally expresses little to no STn and a SKOV3 cell line that stably overexpresses ST6GALNAC1 resulting in increased levels of STn [4] with the human anti-STn-ADC. The human anti-STN-ADC had no significant effect on the SKOV3^CTL^ line but displayed a dose-dependent impact on the SKOV3-ST6GALNAC1 line as determined by an MTT assay. This result agrees with our murine antibody specificity in an in vitro setting using long-term established ovarian cancer cell lines which displayed a range of cells positive for STn [5, 6]. To augment these findings, we assessed the effect of our anti-STn-ADC on four PDO lines three of which were shown to be resistant to one or more drug treatments and one of which was still platinum sensitive [27]. Similar to what we observed in the established OvCa line using the murine anti-STn-ADC, the humanized anti-STn-ADC proved effective in patient derived OvCa organoids, further supporting our argument. The pattern of response was reflected by their STn levels with the 17–121 PDO showing the highest level of STn being the most responsive.

Moving forward, it was essential to determine the predictive power of STn expression relative to the response to the anti-STn-ADC treatment in in vivo models. Similar to that observed with the STn positive and negative SKOV3 cells, the PDX models indicate that the levels of STn impacts the effect of the treatment with anti-STn-ADC. The PDXs derived from the tumor that had relatively low levels of STn responded to anti-STn-ADC, although modestly compared to the PDX hosting the tumor with higher levels of STn positive tumor cells which responded more robustly. These data suggest that not all tumors would equally benefit from the anti-STn-ADC treatment and would require some level of screening for STn expression to enrich for potential responders. This finding supports the conclusions of a previous vaccine trial (Theratope) where it was determined retrospectively that the ability to enrich for patients whose tumors displayed higher levels would likely improve the chance of success [7]. However, the longitudinal expression of STn was not studied in this cohort and it may be dynamic, and/or change in response to treatment and will need to be investigated.

Using a previously described PDX mouse model [25] that mimics, in part, recurrent or resurgence of cancer, we compared a standard carboplatin and paclitaxel regimen against anti-STn-ADC treatment alone and in combination. Interestingly, treatment with single-agent anti-STn-ADC resulted in an improved response over carboplatin and paclitaxel alone in the PDX that hosted the tumor with relatively high levels of STn. We appreciate the PDX model used herein reflects a more acute response rather than a long-term response. Stopping the carboplatin and paclitaxel treatment was meant to mimic tumor resurgence, as shown in the past [25]. More specifically, this model enables us to have some insight if another drug was added at the point the cytotoxic was withdrawn or if a combination strategy was attempted at the onset and the cytotoxic agent was stopped, but the other drug regimen was maintained, was it sufficient to prevent the resurgence prompted by the withdrawal of the cytotoxic regimen. Therefore, we examined two combination strategies with anti-STn-ADC and chemotherapy treatment. The first was concurrent carboplatin and paclitaxel, and anti-STn-ADC, and the second was sequential treatment starting with chemotherapy followed by anti-STn-ADC. We postulated that chemotherapy would enrich for STn positive cells suggesting the sequential strategy would be more effective than the single-agent treatment. This, however, was not the case in our study, where both single-agent anti-STn-ADC and sequential treatments were equally effective. Based on this outcome, we postulate that an anti-STn-ADC might serve as a maintenance strategy; additional studies would bolster or refute this observation.

Historically, other STn based therapies have been less than effective in clinical trials. The Theratope vaccine was tested in breast cancer patients; however, the results were underwhelming. As mentioned, these lackluster results were likely due, in part, to the lack of patient stratification based on their tumor levels of STn. Retrospective ad hoc studies demonstrated that when the patients were divided into those who had detectable levels of STn fared better than those that didn’t have any detectable levels of STn [7]. These results, along with our own observations [4–6], prompted us to determine whether treatment using our anti-STn-ADC would benefit from patient stratification by STn expression. Consequently, we focused on developing a serum ELISA that may serve to identify patients with circulating STn and if the levels were reflective of what was observed in the tumor.

Several antibodies have been used to assess circulating STn levels or detect malignancies with varied outcomes [12, 35–37]. Some of the variability observed in previous studies was likely due, in part, to differences in methodology, the lack of antibody specificity, and/or assessment of STn in the cytoplasm, membrane bound proteins or circulating proteins. We previously demonstrated that some of the historical anti-STn antibodies bound additional glycans and/or may have glycoprotein preferences for antigen recognition [6]. Commercial STn serum quantification assays often utilize CC49 and B72.3 historical antibody pairs, which are not strictly STn specific. We postulated that an assay utilizing our unique anti-STn antibodies might improve assay sensitivity and specificity for future biomarker or patient stratification serum ELISA assays. Based on preliminary non-published data, a subset of anti-STn antibodies were chosen for sandwich ELISA testing, and pairing was guided based upon their light chain CDR sequences. This report described initial development and preliminary characterization of the STn ELISA and explored its suitability to predict clinical response in retrospective OvCa patient cohorts.

As described, we analyzed a cohort of blood samples from patients originally diagnosed with benign or malignant gynecologic disease/conditions. The benign cases were preferentially chosen for their high propensity for a false positive read if CA-125 were the sole discriminating factor. These benign GYN cases had diagnoses such as endometrioma, endometriosis, cystic follicles, leiomyomata, etc. The malignant cases primarily focused on serous ovarian but included a limited cohort of ovarian mucinous, clear cell, and endometrioid cancers. One of our objectives was to determine if the ELISA could detect circulating levels of STn and whether the levels were concordant with STn positivity on FFPE sections from paired samples. For this, we identified a subset of matching samples to determine if there was any similarity between blood and tumor levels.

We did not expect a 1:1 correlation, given some proteins or peptides that present with STn may be on the membrane or are cleaved and found in circulation. For example, MUC16, can be cleaved and the cleaved portion, CA-125 can be found in the circulation. Other STn positive proteins can be internalized or remain on the surface. Others have also suggested that STn can be ubiquitously expressed or irregularly distributed across tumors [6, 7, 9]. Still, others have reported that more aggressive areas might display increased levels of STn [7]. If this were the case, the FFPE sampling might be an issue. However, this was not the case. In the cases we evaluated the majority were concordant, meaning that if STn was high in the serum, it was also highly expressed on the tumor sections with adequate sampling and vice versa. This finding suggests that the ELISA or IHC may serve to triage patients appropriately. Combining the two methods, ELISA and IHC could provide additional confidence.

Unlike others have reported [12, 33], using our antibody combination in a sandwich ELISA we did not see a clear increase in STn levels concordant with the stage of disease except for ovarian clear cell, but this was only a small cohort. We attribute this difference to the specificity of our antibody cocktail used in our ELISA as opposed to the antibodies used by others, many of which we showed were not specific to STn [6].

Given that our STn ELISA was generally able to distinguish between malignant and non-malignant cases, we were curious how it compared to CA-125 and if it augmented CA-125’s ability to detect ovarian cancer. CA-125 has been FDA approved to monitor ovarian cancer patients’ response to treatment. CA-125 has ∽ 90% specificity and 50–60% sensitivity [38, 39]. However, CA-125 is not approved as a diagnostic measure in ovarian cancer due to its low specificity since it is found elevated in benign conditions such as endometriosis, ovarian cysts, and fibroids [40–44]. Despite introducing new biomarkers, such as HE4, and other multi-modal tests (ROMA, ROCA, OVA1, Overa), CA-125 [45] is still often one of the key biomarkers studied. CA-125 is a mucin-type glycoprotein product of cleaved MUC16. The extracellular domain of CA-125 is rich in N- and O-liked glycans with 249 potential N-glycosylation and over 3,700 O-glycosylation sites [46]. CA-125 glycan post-translational modifications often differ between normal and malignant conditions, expressing truncated glycans in OvCa such as the STn [47]. The Elecsys CA 125 II clinical assay does not allow discrimination among various glycoforms of CA-125 as neither mAb clone is significantly influenced by N- or O-glycosylation of CA-125 [48]. We postulated the analysis of post-translational modifications such as glycosylation may increase the specificity and sensitivity of existing OvCa biomarkers or even lead to novel independent biomarkers with improved accuracy. Over 70% of human blood proteins are glycosylated, and these modifications often play important roles in adhesion, migration, immune recognition, and other signaling pathways [49]. However, despite the increased specificity of the STn antibody and the number of glycoproteins with STn expressed, we were unable to demonstrate improvement over CA-125 independently or augment its ability to detect ovarian cancer except for the mucinous clear cell cancers. Despite showing statistical differences, the small sample size warrants caution about its value in augmenting CA-125. Together, these findings suggest that using an antibody-based approach against more than one of the aberrant glycosylations associated with malignancies might be a better approach to improve diagnostic potential.

## Conclusion

Collectively, we conclude our preclinical studies, along with others, support the concept that targeting STn with a highly specific antibody-drug conjugate where there is evidence of STn present in either circulation and/or the tumor, might be a viable clinical option for treatment of OvCa. Further studies are needed to validate whether an anti-STn-ADC could be used as maintenance strategy.

### Electronic supplementary material

Below is the link to the electronic supplementary material.

Supplementary Material 1

Supplementary Material 2

Supplementary Material 3

Supplementary Material 4

Supplementary Material 5

Supplementary Material 6

Supplementary Material 7

Supplementary Material 8

## Data Availability

The datasets used and analyzed during the current study are available from the corresponding author on reasonable request. Sharing of primary human tumor material are subject to approval by institutional review board, institutional data and tissue management and sharing committees, and approved material transfer agreements.
