# Supplementary material for: Sialyl-Tn serves as a potential therapeutic target for ovarian cancer
Source: J Ovarian Res. 2024 Apr 2;17:71. doi: 10.1186/s13048-024-01397-1 (PMC10985924; doi:10.1186/s13048-024-01397-1)
Supplement: Supplementary file 1 — Supplementary Material 1 [file 13048_2024_1397_MOESM1_ESM.pdf]

Cancer Subtype				
Benign	Clear	Endometrioid	Mucinous	Serous
146	37	29	20	188

Cancer Stage			
I	II	III	IV
69	35	123	45

Age Range			
<30	30-50	50-70	>=70
9	106	225	80
