# Supplementary material for: Sialyl-Tn serves as a potential therapeutic target for ovarian cancer
Source: J Ovarian Res. 2024 Apr 2;17:71. doi: 10.1186/s13048-024-01397-1 (PMC10985924; doi:10.1186/s13048-024-01397-1)
Supplement: Supplementary file 2 — Supplementary Material 2 [file 13048_2024_1397_MOESM2_ESM.pdf]

IHC Score	Code	Subtype	Serum STn	CA125	Grade	Stage
3	LAA1, 66, 153	Serous	2.760704322	10853	3	IIIC
0	LAA6	Serous	0.026551936	604.4	3	IIIC
2	LAA16, 171	Serous	0.333990403	308	3	IV
1	LAA17, 177	Serous	0	122.6	3	IIIC
0	LAA18, 187	Serous	0.037859645	185	3	IIIC
1	LAA19, 193	Serous	0.023074048	568.7	3	IIIC
0	LAA22, 201	Serous	0	130.4	2	IIIC
1	LAA23, 203	Serous	0.081115207	133	3	IIIC
0	LAA25, 142	Serous	0	2089	3	IV
3	LAA26, 213	Serous	0.353647098	743.6	3	IIIA
0	LAA27, 214	Serous	0.041931778	9	3	IIIC
0	LAA28, 216	Serous	0	173	3	IIIC
3	LAA29, 217	Serous	0.425992424	180.1	3	IIIC
0	LAA30, 218	Serous	0.015484848	246.3	3	IIIC
1	LAA32, 228	Serous	0.057685636	83	3	IIIC
2	LAA33, 229	Serous	0.576730297	269.5	3	IIIC
2	LAA34, 231	Serous	0.487077965	3624	3	IIIC
0	LAA35, 234	Serous	0.03525	528	3	IIIC
1	LAA40, 151	Serous	0.032555489	846	3	IIIC
2	LAA76	Serous	0.410678392	186.3	3	IIIC
0	LAA82	Serous	0	344.4	3	IIIC
3	LAA85	Serous	2.187146474	988	2	IV
1	LAA95	Serous	0	989.5	2	IIIC
2	LAA103	Serous	1.304360978	9603	3	IV
0	LAA107	Serous	0.909100076	2301	3	IIIC
0	LAA123	Serous	0	76.1	2	IIIC
3	LAA128	Serous	0.639591175	1807	3	IV
2	LAA130	Serous	0.430984762	765	3	IIIC
0	LAA131	Serous	0	2537	3	IIIC
0	LAA133	Serous	0.025217194	84	3	IV
3	LAA139	Serous	1.948097437	228	3	IIIC
0	LAA158	Serous	0.331790441	612.4	3	IIC
2	LAA166	Serous	0.356001294	158.7	3	IIIC
1	LAA180	Serous	0.662772761	284	3	IIIC
0	LAA185, 385	Serous	0.032973389	267	1	IV
3	LAA186	Serous	2.028021766	1675	3	IA
2	LAA196	Serous	0.231969163	4369	3	IIC
0	LAA202	Serous	0.027883472	99.4	3	IIIC
3	LAA202	Serous	0.027883472	99.4	3	IIIC
2	LAA 632	Serous	0.730286025	3914	3	IIIC

0	LAA410	Clear Cell	0.135443038	520	N/A	IIIC
1	LAA414	Clear Cell	0.088970758	783.8	3	IIC
2	LAA415	Clear Cell	0.127653696	108.3	3	IIIA
0	LAA418	Clear Cell	0.273359429	125	2	IIIC
3	LAA419	Clear Cell	0.125504643	348	N/A	IIIC
2	LAA421	Clear Cell	0.166336634	542.6	3	IIIA
0	LAA425	Clear Cell	0.136249904	50	3	IC
1	LAA427	Clear Cell	0.079350833	51.9	3	IC
0	LAA429	Clear Cell	0.340038114	1112	3	IV
1	LAA430	Clear Cell	0.092260404	133	3	IIB
1	LAA432	Clear Cell	0.099145509	446	3	IIIC
0	LAA433	Mucinous	0.115668203	207.6	2	IIC
1	LAA441	Mucinous	0.104147465	31	1	IA
1	LAA443	Mucinous	0.049308756	15.1	2	IA
2	LAA446	Mucinous	0.121198157	13	1	IC
1	LAA447	Mucinous	0.455379747	612	2	IA
1	LAA448	Mucinous	0.103270042	34.8	2	IA
2	LAA449	Mucinous	0.131751055	75	1	IA
1	LAA453	Mucinous	0.149156118	12	1	IA
1	LAA478	Endometrioid	0.079774855	13.9	1	IIA
1	LAA482	Endometrioid	0.093309081	13.5	2	IB
