# Supplementary figures and images for: Sialyl-Tn serves as a potential therapeutic target for ovarian cancer
Source: J Ovarian Res. 2024 Apr 2;17:71. doi: 10.1186/s13048-024-01397-1 (PMC10985924; doi:10.1186/s13048-024-01397-1)

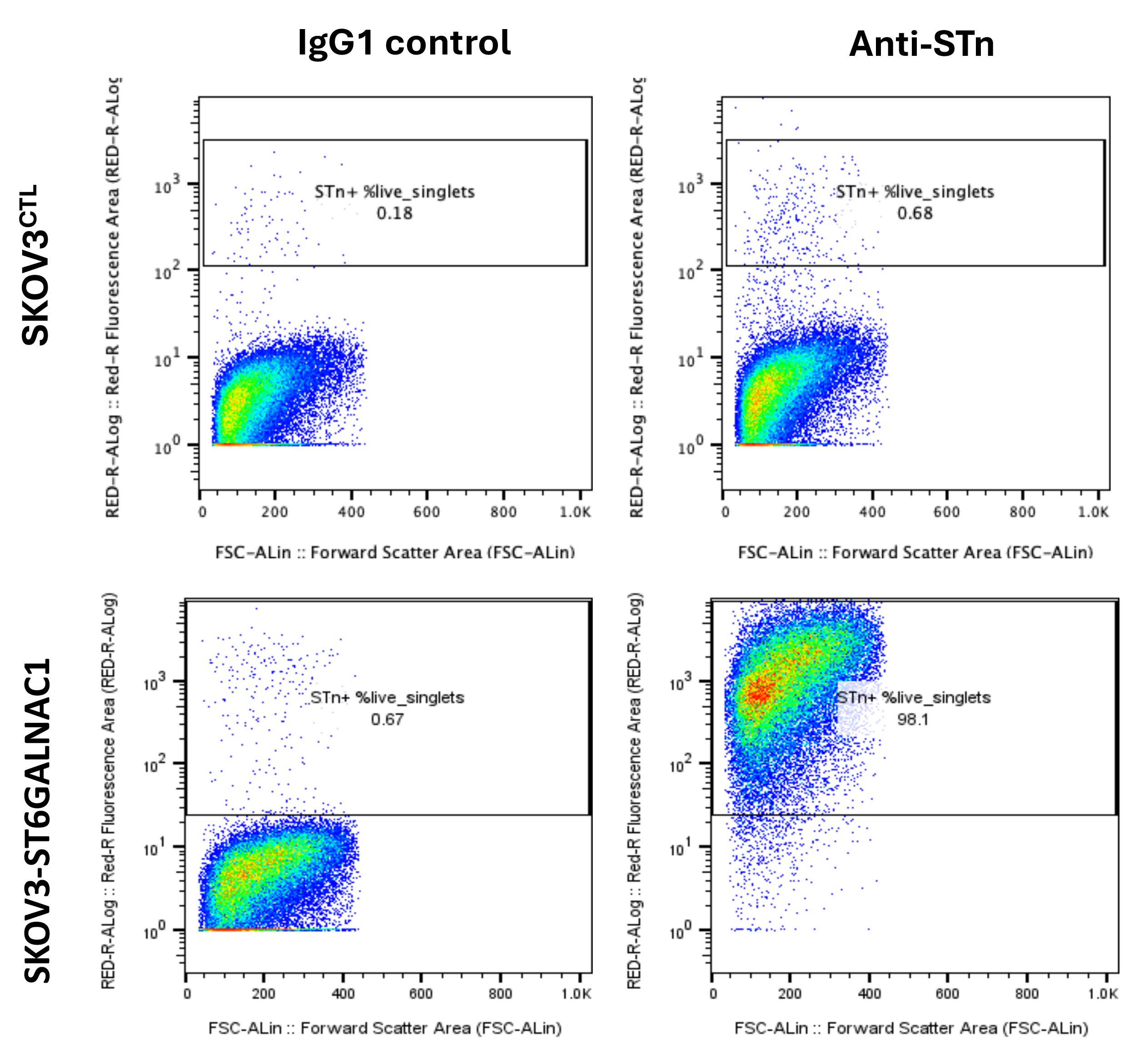

Supplement: Supplementary file 3 — Supplementary Material 3 [file 13048_2024_1397_MOESM3_ESM.tif]

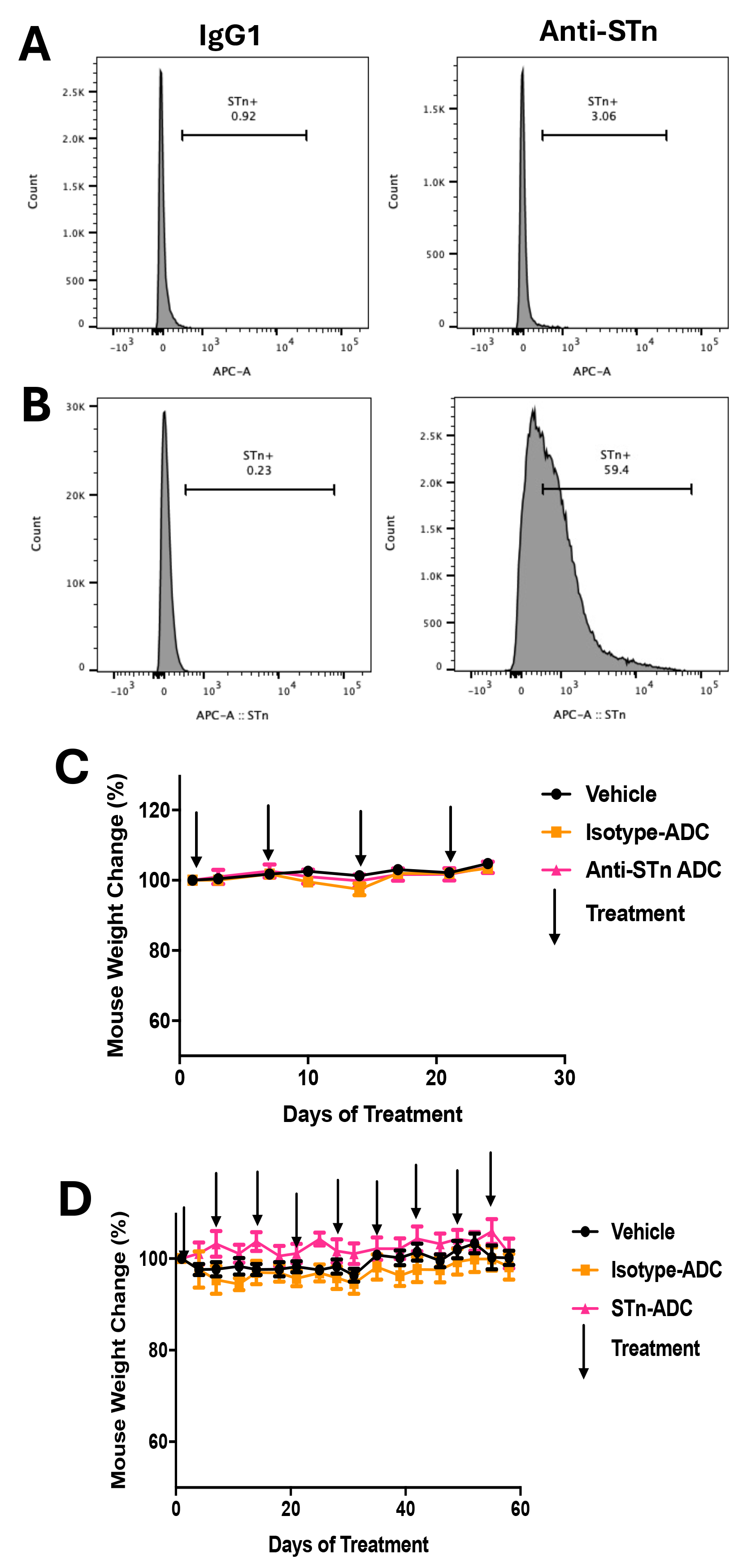

Supplement: Supplementary file 4 — Supplementary Material 4 [file 13048_2024_1397_MOESM4_ESM.tif]

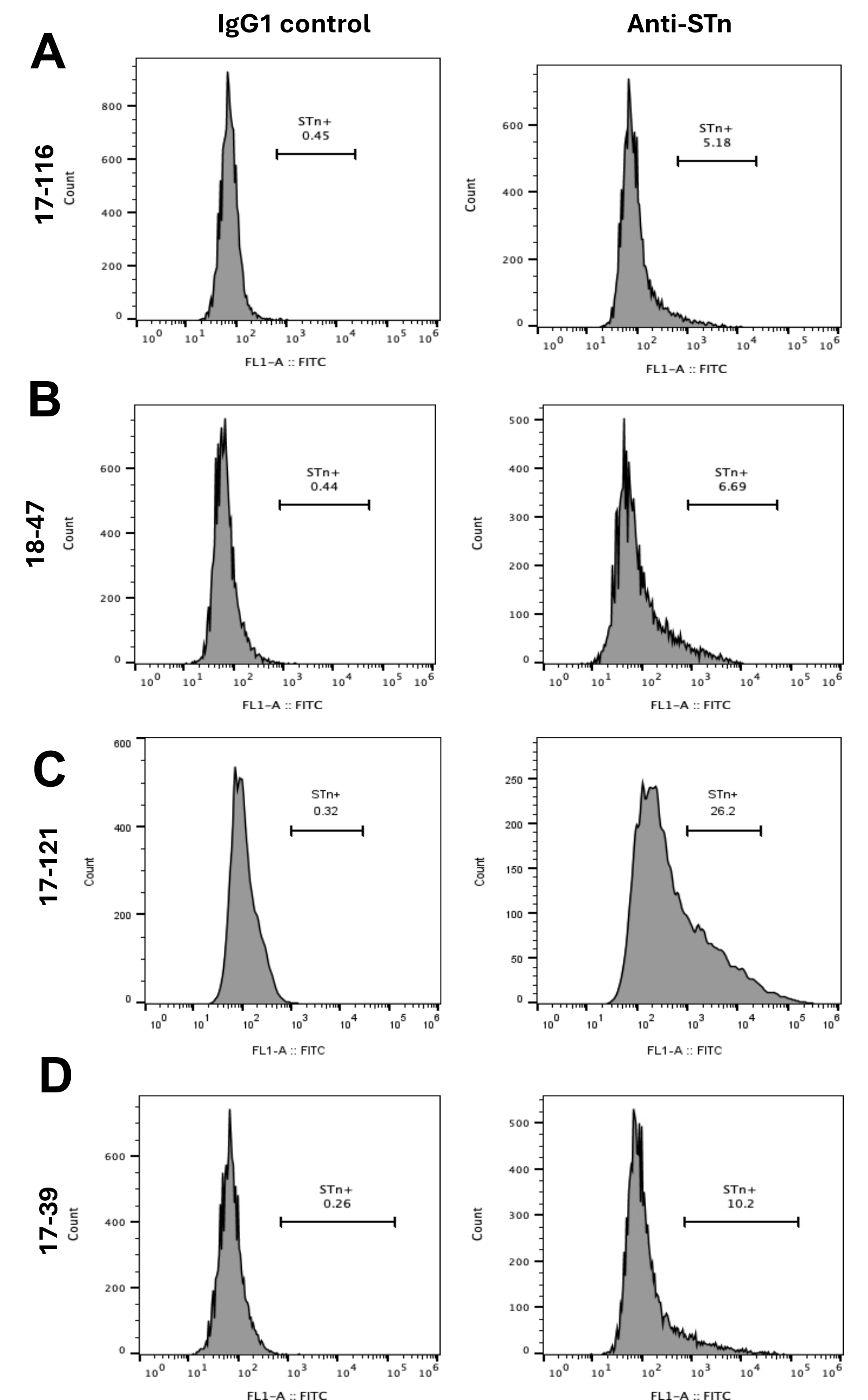

Supplement: Supplementary file 5 — Supplementary Material 5 [file 13048_2024_1397_MOESM5_ESM.tif]

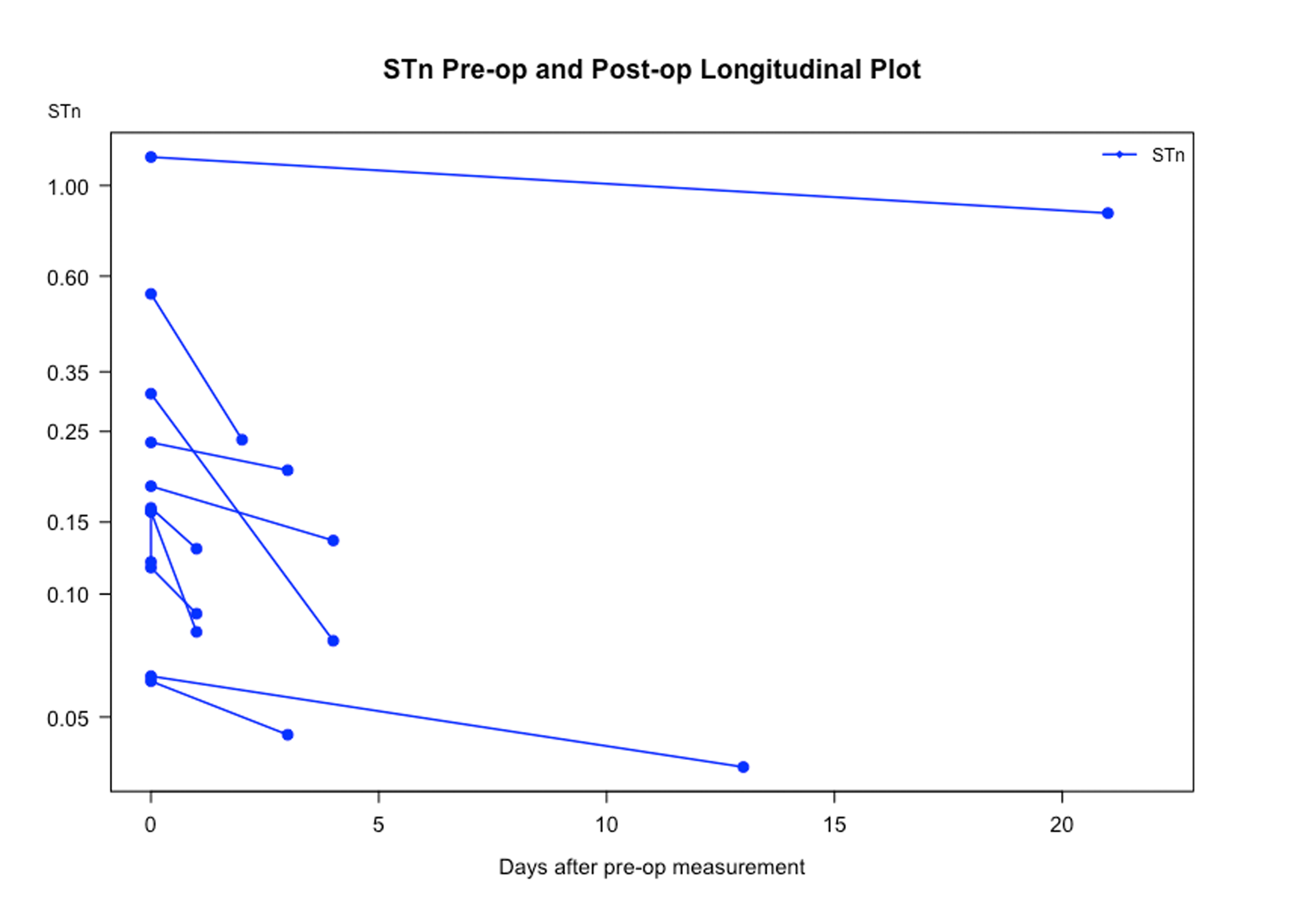

Supplement: Supplementary file 7 — Supplementary Material 7 [file 13048_2024_1397_MOESM7_ESM.tif]
